# Antibiotic-Therapy-Induced Gut Dysbiosis Affecting Gut Microbiota—Brain Axis and Cognition: Restoration by Intake of Probiotics and Synbiotics

**DOI:** 10.3390/ijms24043074

**Published:** 2023-02-04

**Authors:** Divakar Dahiya, Poonam Singh Nigam

**Affiliations:** 1 Wexham Park Hospital, Wexham Street, Slough SL2 4HL, UK; 2 Biomedical Sciences Research Institute, Ulster University, Coleraine BT52 1SA, UK

**Keywords:** antibiotics, dysbiosis, microbiota, gut, brain, health, probiotics, synbiotics, psychobiotics, food, beverage, nutrition

## Abstract

Antibiotic therapy through short-term or repeated long-term prescriptions can have several damaging effects on the normal microbiota of the gastrointestinal tract. Changes in microbiota could be multiple including decreased diversity of species in gut microbiota, changed metabolic activity, and the occurrence of antibiotic-resistant strains. Antibiotic-induced gut dysbiosis in turn can induce antibiotic-associated diarrhoea and recurrent infections caused by *Clostridioides difficile*. There is also evidence that the use of different chemical classes of antibiotics for the treatment of a variety of ailments can lead to several health issues including gastrointestinal, immunologic, and neurocognitive conditions. This review discusses gut dysbiosis, its symptoms and one important cause, which is antibiotic therapy for the induction of gut dysbiosis. Since the maintenance of good gut health is important for the well-being and functioning of physiological and cognitive activities through the normal gut-microbiota–brain relationship, the condition of dysbiosis is not desirable. Specific therapies are prescribed by medical practitioners for the cure of a variety of ailments, and, if the prescription of antibiotics becomes unavoidable, there is a possibility of the onset of gut dysbiosis as the side or after effects. Therefore, the restoration of imbalanced gut microbiota to its balanced condition becomes necessary. A healthy relationship between gut microbiota and the brain can be achieved with the introduction of probiotic strains into the gut in a practical and consumer-friendly way, such as consumption of food and beverages prepared with the use of characterised probiotic species, fermented foods as the potential biotics, or synbiotic supplements.

## 1. Introduction

The gut microbiota of a healthy individual is normally comprised of harmless species of bacteria. The contribution of the gut microbiota to human health has been established in that the normal gut microbiota provides protection against invasive microorganisms, which could be potential pathogens. The intestinal microbiota is a dynamic community of millions of microorganisms that play important roles in the sustainability of human health [1]. Most of these microbial species present in gastrointestinal tract have a positive effect on the host’s health and contribute to the natural physiological processes through the production of their metabolites [2]. A healthy gut microbiome represents a highly diverse community, estimated to exceed 10^14^ microorganisms, with functions ranging from carbohydrate fermentation and vitamin synthesis to immune system development, as well as its effects on the functionality of the nervous system [3,4,5]. The host possessing a balanced normal gut microbiota is benefitted with the protection against disease-causing microbial species, by means of a power of colonisation resistance [6].

Nonetheless, therapy with the medication of antibiotics of a complex chemical nature can disrupt the microbial balance of the gut, which results in the development of antimicrobial resistance in pathogens and other diseases due to the after effects of chemotherapy. When the gut microbiota is interrupted and becomes out of balance, it can lead to dysbiosis. When the normal human microbiota in the gastrointestinal tract, including small and large intestines, are affected by harmful microbial species, a condition of dysbiosis typically occurs. This might occur through the disruption in colonisation resistance in a number of ways, such as direct or side effects on normal gut microbiota by the intake of medication, competition between pathogens and gut microbiota for the available nutrients in the gut, and eventually a decline in the host’s immune responses [7].

The reason for the disturbance in healthy microbiota could be short- or long-term treatments with the oral consumption of antibiotics. In the general practice of chemotherapy, a large group of chemicals is used as antibiotics for the treatment of different types of infections developed in different organs and systems of patients. The clinical use of antibiotics has modernised the scope of medicine dealing with the treatment of various infectious diseases common in diverse communities in all countries. Nevertheless, their therapeutic actions are not limited to the disease-causing pathogens for which they are prescribed, and hence these chemicals generally also influence the sustainability of beneficial microbes present in the gut of patients. The commonly prescribed antibiotics belong to several classes based on their chemical structures, including beta-lactam antibiotics, aminoglycosides, daptomycin, fluoroquinolones, glycopeptides (teicoplanin and vancomycin) and linezolid.

Antibiotics cannot perform their preferential action on pathogens specifically, but also act on all microorganisms including the normal microbiota present at that time in the gut of a patient. Such activity of antibiotics affects the constitution of normal gut microbiota. The course of antibiotic therapy adversely affects the diversity of the microbiota in the gastrointestinal tract. As a result, antibiotics interfere with host–microbes interactions, and immune system homeostasis, and impair colonisation resistance toward new pathogenic strains entering the host system. In fact, treatments using antibiotics for targeting infections can also impair the severity of other ailments, such as inflammatory bowel diseases and colitis through the disturbance of toll-like receptor signalling. As a result, variations in the diversity of gut microbiota occur, which in the longer term could be linked to a number of health conditions, including cardiovascular disease [8,9], liver disease [10,11], obesity, diabetes, etc. [12,13,14].

In further sections, this review will discuss gut dysbiosis, its symptoms, types of antibiotics (prescribed for the treatment of different health conditions) causing dysbiosis, cognitive weakening with the changes in molecular level communication between gut microbiota and brain, and finally the restoration of gut-microbiota imbalance to eliminate the condition of dysbiosis.

## 2. Gut Dysbiosis

The term dysbiosis has been described as the disruption of the symbiotic balance between microbiota and the host. Dysbiosis implies frequent changes and eventually leads to disturbances in the ecology of gut microbiota, which has been characterised as an adaptation in the composition and functioning of the microbiota. Dysbiosis can be caused by mainly two factors, which could be either environmental or host-related [15]. The condition of dysbiosis overpowers the resistance and protection capabilities of the microbial ecosystem in the gut [16]. Alterations in gut microbiota are considered to be associated with the pathogenesis of several non-communicable diseases; subsequently, an uncontrolled dysbiosis could lead to the transition of treatable ailment conditions to the level of chronicity in the long term. Dysbiosis is a risk factor for certain diseases found to be closely associated with health conditions, including gastrointestinal diseases like colitis, coeliac disease, bowel disorders (IBS and IBD), leaky gut syndrome, liver disease, cancer in colon or rectum, *Candida* yeast infection, diabetes, and skin conditions, such as eczema, etc. Antibiotic-induced gut dysbiosis in turn can induce diarrhoea and recurrent infections caused by *Clostridioides difficile* [2,17,18].

### Dysbiosis Symptoms

Before the actual diagnosis of a specific health condition, certain symptoms can initially indicate the actual occurrence of dysbiosis in a patient. The symptoms will be obvious and different depending on the location of the imbalance in the gut microbiota. Dysbiosis symptoms will also vary based on the type of gut bacteria affected. Common symptoms of dysbiosis include diarrhoea, bloating, bad breath (halitosis), fatigue due to mal-absorption of nutrients by an upset digestion, constipation, nausea, chest pain, and vaginal or rectal itching, etc. [19]. Symptoms may also include cognitive weakening such as depression, anxiety, and having difficulty in thinking or concentrating. Prolonged dysbiosis induced by antibiotic consumption can weaken the host’s immune system [20]. The cognitive weakening could be due to the effect of dysbiosis on gut microbiota–brain communication [21]. Therefore, it is important to understand the side or after effects of antibiotic therapy on the relative survival of normal gut microbiota with pathogenic species of microorganisms, and opportunistic bacterial species present in the gut.

## 3. Antibiotic-Therapy-Induced Gut Dysbiosis

Antibiotics are major disruptors of gut microbiota [22]. The oral use of antibiotics releases chemicals into the gut system causing a disturbance in the intestinal microbiota, which could be through the interactions between the normal gut microbiota and opportunistic and pathogenic bacteria present in the gut [23]. This leads to an increase in the risk of colonisation by intestinal pathogens. Still, the understanding of the effect of antibiotics is limited on potentially beneficial and pathogenic bacteria under the conditions of gut microbiota dysbiosis. The effects of antibiotics on the gut microbiota could be temporary or permanent depending on the type of medication prescribed (Table 1), and the duration of antibiotic treatment [24].

During and after the long-term therapy with the prescription of different classes of antibiotics (Table 2), the delicate balance of the gut microbial community can be disrupted, leading to detrimental effects on the patient’s health conditions, such as enhanced susceptibility to other microbial infections due to the loss of immunity, development of gut inflammation, as well as an effect on the mental health of the patient.

In a recent report published in 2022, 16S rRNA gene sequencing was used to evaluate the short- and long-term effects of four different commonly used antibiotics, including ampicillin, vancomycin, metronidazole and neomycin on the murine intestinal microbiota. The researchers found changes in the intestinal microbiota, which indicated the impact of antibiotics’ action. As a result, dysbiosis of the gut’s normal microbiota led to a struggle for their sustainability among different bacterial populations in the gut. The studies identified the consistent changes in the microbiota following chemotherapy with the use of quinolone and metronidazole compounds, and reported that the combination treatment with a mixture of antibiotics may even result in a long-term dysbiosis, compared with the treatment with one type of antibiotic [68].

## 4. Cognitive Weakening with Changes in Molecular Level Communication between Gut Microbiota and Brain

The role of gut microbiota has been identified and studied for its involvement in brain functions, including cognition, brain performance [69,70], cognitive weakening and mental health [71,72]. The composition and diversity of the gut microbiome are affected by variations in gut health, which could be the result of several factors, mainly change in diet, malnutrition, or infections in the gastrointestinal tract and their treatment over a period of medication prescription. Gut bacteria play an important role in digestion and immune responses, and in the regulation of entero-endocrine signalling pathways. Through the review of recent reports, information can be accessed on the gut microbiota, gut–brain axis, hypothalamic pituitary adrenal axis, neuropsychiatric disorders (such as autism, depression and schizophrenia), and cognitive behaviour [21,73].

It is interesting to know that resident microbial strains in the gut have an impact on the production of major neurotransmitters, such as dopamine, norepinephrine, also called noradrenaline, serotonin [74], histamine and γ-aminobutyric acid [75]. The communication between the gut and brain through the vagus nerve, playing a role in cognition to stress response, has been reviewed for its important role in mental health [21]. The molecular level communicating relationship between the diversity and composition of gut microbiota and brain function are programmed through the microbiota-gut–brain axis [76].

Signals are transported from the gastrointestinal tract (GIT) and its microbiota to the brain through the network of fibres of the afferent vagus nerve, linked to receptors in different parts of the digestive system, including oesophagus, liver and pancreas [77]. After receiving these stimuli, the brain sends a response through the fibres of the efferent vagus nerve to entero-epithelial cells. Since the fibres of the vagus nerve are not in direct contact with intestinal microbiota and the wall of GIT, all signals from the brain reach the gut microbiota via millions of neurons from the enteric nervous system in the submucosa and myenteric plexus of the gut wall [78]. The adjustment, development and renewal of neurons in the enteric nervous system are controlled by specific strains of gut microbiota, which have the ability to produce and metabolise hormones. Signals generated by the hypothalamus reach the pituitary and adrenal glands. In this way, the communications are established, with entero-epithelial cells passing through the hypothalamic pituitary adrenal axis. Gut bacteria produce short-chain fatty acids (SCFA) [79], which stick to free fatty acid receptors on the surface of intestinal epithelial cells for their interaction with neurons. Reports have indicated that gut bacteria are involved in altering the synthesis and degradation of neuro-transmitters [80], and the functioning of the brain [81].

The disturbances in the GIT microbiota have been broadly associated with symptoms of major depression disorders, although the identity of causal microbial species and the underlying mechanisms are yet to be fully understood. The effects of short-term antibiotic-induced depletion or changes in the microbiota have been examined on cognitive function in mice. The work of Fröhlich et al. has been considered a basis for translational research into the role of gut microbiota in human cognition. The authors depicted a strong effect mediated by antibiotics on GIT microbiota composition. In this study, change in cognitive behaviour was found linked with the unusual levels of peripheral metabolites and functional changes in neural signalling pathways in the brain [82].

*Bacteroides* species have been found to differentially modulate depression-like behaviour via gut–brain metabolic signalling. In a recent report of 2022, a team of researchers identified *Bacteroides* species that contributed to depression susceptibility in mice by metabolic regulation along the gut–brain axis. The report highlighted activity in three strains of *Bacteroides, B. uniformis*, *B. fragilis,* and *B. caccae*, which impaired the hippocampal neurogenesis. The hippocampal serotonin levels were found adversely affected by these species of *Bacteroides* [83].

The administration of antibiotics can be a useful pharmacological tool to investigate the contributory relationships between fluctuations in gut microbiota, corresponding to brain function and cognitive behaviour. A molecular-level understanding of the depletion of GIT microbiota acutely or chronically was studied through the administration of a mixture of antibiotics at different stages of the lifecycle of animals. This information would be useful in allowing the dose of antibiotics and simulating the clinical condition in humans [84]. A persistent procedural concern related to the administration of antibiotics implicates the pharmacokinetic properties of the antibiotics used. A particular type of chemical structure (Table 1 and Table 2) with high bioavailability would enter the blood circulation, and might cross the blood–brain barrier, with a possible direct effect on the central nervous system. In a study, the test antibiotics included ampicillin, bacitracin, meropenem, neomycin and vancomycin, whereas only ampicillin was found absorbed to a very low extent from the human gut [85].

In the case of cognitive impairment (confirmed in a test for the disruption of novel object recognition memory), the measure of antibiotic absorbed was found below the detection limit. The researchers concluded the cognitive impairment in antibiotic-treated mice must have resulted from gut dysbiosis rather than from a systemic response from the administration of an antibiotic. This report on studies performed on antibiotic-treated mice discussed several important aspects, such as—multiple antibiotic treatments cause dysbiosis and metabolite depletion in the gut; circulating metabolites may be messengers in the communication between gut dysbiosis and brain; antibiotic-induced dysbiosis impairs cognitive performance; cognitive impairment due to gut dysbiosis is associated with changes in the expression of tight-junction-proteins, brain-derived neurotrophic factor, and the serotonin transporter, etc. [82]. 

## 5. Restoration of Gut-Microbiota Imbalance

Considering the fact that short-term broad-spectrum antibiotic treatment given orally intensely affects the phenotype and function of mucosal immune cell populations that occur by induction of inflammatory activation of colonic iNKT and T helper cells, then the implications of repeated broad-spectrum antibiotic use could be further critical and the cause of the breakdown of acceptance mechanisms between the normal microbiota resident in the gastrointestinal tract and the immune system of the host. Antibiotic treatment given to younger patients in their early years of life will lead to an increased risk of gut inflammatory conditions. Hence, the condition of dysbiosis needs to be rectified at its early detection to restore the important role of gut microbiota in the modulation of intestinal immunity. The intake of diet is an important resource and practical for correcting the bacterial imbalance in the gut, therefore the addition of pre-, pro-, and synbiotic food and beverages in nutrition can support keeping the gut microbiota in a balanced condition [86,87]. Several commercial supplements are also available that contain specific QPS (Qualified Presumption of Safety) strains of probiotic bacteria. A particular synbiotic formulation can be taken by consumers for a required health benefit, as different supplements have been designed to meet the specific needs of consumers [2,21,88].

Dysbiosis could be repaired with the re-introduction of friendly microbial species in form of important probiotics in the gut. Consumption of food or beverages that contain live probiotic bacteria at the time of consumption would help in the correction of dysbiosis [89,90]. Characterised QPS strains are well known for their ability to modulate the mucosal immune system, which benefits the host’s well-being. The intake of probiotic foods, providing the sustainability of a healthy gut microbiota, can also manipulate the functioning of the gut–brain axis. Probiotic bacteria have been examined for their significant impact on homeostasis, inflammatory response and immunopathology suppression [91,92,93].

**Figure 1:**
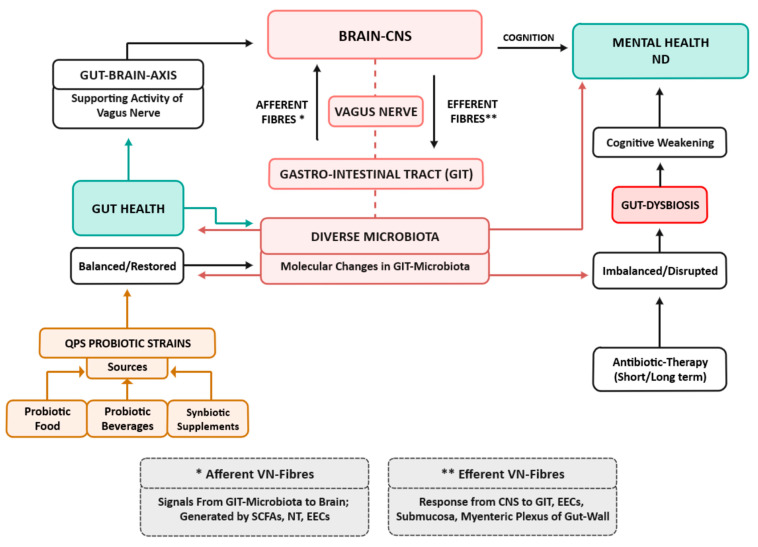
Antibiotic-therapy-induced gut dysbiosis affecting gut-microbiota–brain axis and cognition, and its restoration by probiotics [2,21,86,89,90] QPS—Qualified presumption of safety; CNS—Central nervous system; ND—Neuropsychiatric disorders; NT—Neurotransmitters; SCFA—Short-chain fatty acids; EEC—Entero-epithelial cells.

The potential for the consumption of functional foods has also been explored to help in the remediation of certain psychological issues. Studies have reported that correction can be made in the composition of imbalanced gut microbiota. The biotherapy in the form of ingestion of bioactive components including prebiotics, postbiotics, and parabiotics in fermented foods can affect the intelligence, mood, autism, behaviour and psychology of its host through the gut–brain axis (Figure 1) [94,95,96].

Prebiotics and oligosaccharide-based fibres are selectively utilised as substrates available in the host’s gut system by probiotic strains to develop and sustain their population in the gut [97,98]. A healthy balance of gut microbiota restored and maintained with the consumption of probiotic products plays an essential role in health improvement, infection control, and disease treatment and management [99,100,101]. A balanced microbiota senses and overtakes the occurrence of opportunistic and pathogenic strains providing protection from the onset of dysbiosis, thereby preventing the condition of several health issues [94,102,103,104].

The exopolysaccharides secreted by *Lactobacillus reuteri* (current nomenclature—*Limosilactobacillus reuteri*), and the immune-modulatory effect of this probiotic strain have been investigated on an Epithelial Cell Line IPEC-J2 [105]. Cell lines were challenged with a primary enteric pathogen, *Salmonella typhimurium,* which infects both humans and animals. This pathogen most often causes gastroenteritis, provoking the condition of dysbiosis. The most relevant result of this in vitro study was a case of immunity modulation by the probiotic strain, where *L. reuteri* was found to be a factor in the stimulation of the innate immune-cell response. The response was exhibited by anti- and pro-inflammatory activities. The pretreatment of pathogen-bacterial cells with live *Lactobacilli* or their exopolysaccharide prior to infection showed a suppressive effect on the inflammatory response induced by *Salmonella typhimurium* [106].

## 6. Conclusions and Recommendations

Information collected from the above-discussed research outcomes has proved that the onset of dysbiosis predominantly disturbs the balance of microbial diversity and their numbers in gut microbiota. One of the several causes of dysbiosis has been identified as the side or after effects of chemotherapy prescribing different classes of antibiotics. It is suggested that, after antibiotic treatment, the analysis of the diversity of gut microbiota and its differential abundance should be performed. It is recommended that studies investigating the effect of antibiotics on the gut microbiome should exclude those participants that have been subjected to antibiotic treatment within four months prior to collection and analysis of baseline samples. Additional examinations need to be made into the functional implications of antibiotic-induced dysbiosis in the gut microbiome.

Antibiotics can induce long-lasting unfavourable effects on patients, compromising the diversity, composition and altered functions of the gut microbiota. The effectiveness of different clinical therapies also depends on the action of the gut microbiota due to its condition at the time of treatment. Different antibiotic treatments affect the ability of the gut microbiota to control intestinal inflammation, as it has been studied in cases where faecal microbiota was transplanted in an experimental colitis model, and in ex vivo experiments with human intestinal biopsies. Although some modification strategies to prevent antibiotic-induced dysbiosis have been explored, such as the simultaneous administration of probiotics during antibiotic therapy, or the transplant of autologous faecal microbiota after the course of antibiotic treatment, both approaches have not shown complete success in the restoration of healthy gut microbiota, particularly in cases of acute dysbiosis, or in weaker patients with low immunity [107,108,109]. 

The characterisation of microbiota should be supplemented by the exploration and analysis of the resistome, metagenome, and metabolome to provide more insight into the functional implications of antibiotic therapy on the microbiome. There is a relationship between the pharmacokinetic and pharmacodynamic of antibiotics and their clinical response. The action of chemotherapy includes efficacy as well as the toxicity of chemical molecules of drugs used as antibiotics. This relationship can be applied to define the therapeutic range of a particular class of antibiotic class for the purpose of therapeutic drug monitoring. The critical step is to define exposure to the doses prescribed and it is equally important to prescribe the correct class of antibiotic. Before the commencement of chemotherapy using an antibiotic, the infection-causing pathogens should be identified in clinical testing. Accordingly, a specific type of antibiotic or a broad-spectrum type should be prescribed to target the infections caused by Gram-positive or Gram-negative bacteria, or other microbial infections caused by fungal and yeast pathogens. This strategy of clinical practice can prevent the development of antibiotic resistance in pathogens being treated. Therapeutic drug monitoring should only be regarded as a means to achieve the main goal of providing safe and effective antibiotic therapy for all types of infections [110].

In the last few decades, there has been a rise in the number of studies on gut microbiota [111], and the focus of research has begun to move toward clinical and therapeutic studies to understand how the gut microbiota can influence human health and be effective in the alleviation of several diseases. In conclusion, when the essential gut microbiota is maintained at a specific population level representing normal healthy microbial ecology, there are multi-fold benefits to the host. A healthy gut creates environmental conditions sufficient to inhibit the invasion and virulence of pathogenic species, influences the host’s mucosal immune cell functions, and has its overall impact supporting the physical well-being and mental health of its host.

## Figures and Tables

**Table 1:** Types of antibiotics prescribed for the treatment of different health conditions.

Antibiotics Prescribed	Treatment for Ailments	Reference
Metronidazole	Bacterial vaginosis in HIV-infected female	[25]
Norfloxacin or Ciprofloxacin	Liver cirrhosis	[26]
Levofloxacin	Type 2 diabetic patients scheduled for cataract surgery	[27]
Doxycycline; Roxithromycin	Functional endoscopic sinus surgery for bilateral chronic rhinosinusitis	[28]
Cefuroxime + ceftriaxone	Bilateral bronchopneumonia in 4 to 5-year-old children	[29]
Amoxicillin + furazolidone	*H. pylori*-positive gastritis	[30]
Rifaximin	Decompensated cirrhosis with hyperammonaemia	[31]
Minocycline	Females with acne	[32]
Doxycycline	Acne	[33]
Amoxicillin + metronidazole	Untreated severe periodontitis	[34]
Rifaximin	IBS with diarrhoea	[35]
Azithromycin	Severe chronic asthma	[36]
Amoxicillin	Molar extraction	[37]
Azithromycin	Chronic symptomatic asthma	[38]
Metronidazole with clarithromycin	Gastric or duodenal ulcer	[39]
Tetracycline + metronidazole	*H. pylori*-positive gastritis	[40]
Amoxicillin + ceftazidime	Pre-term infants with suspicion of bacterial infection	[41]
Erythromycin	Non-cystic fibrosis bronchiectasis	[42]
Amoxicillin + clarithromycin	*H. pylori*-positive	[43]
Amoxicillin + metronidazole	Untreated chronic periodontitis	[44]
Ampicillin/sulbactam + cefazolin	Infected pacemaker	[45]
Moxifloxacin	Hospitalised patients (internal medicine)	[46]
Erythromycin	Non-cystic fibrosis bronchiectasis	[47]
Ciprofloxacin; Nitrofurantoin	Urinary tract infection (UTI)	[48]
Levofloxacin	Chronic rhinosinusitis	[49]
Amoxicillin + clarithromycin	*H. pylori-positive* ulcers	[50]
Nitrofurantoin	Uncomplicated UTI	[51]
Amoxicillin + metronidazole	Periodontitis	[52]
Azithromycin	Emphysema	[53]
Amoxicillin + clarithromycin	*H. pylori-positive*	[54]
Amoxicillin + fosfomycin + metronidazole	Ulcerative colitis	[55]
Rifaximin	Decompensated cirrhosis	[56]
Lymecycline	Acne vulgaris	[57]

**Table 2:** Antibiotics studied for changes in the condition of gut microbiota.

Antibiotics Class	Antibiotic Prescribed	Changes in Host Gut	References
Cell-wall-disrupting antibiotics	Beta-lactam and glycopeptide Amoxicillin; Cloxacillin; Ceftazidime; Ceftriaxone	Beta-lactam and glycopeptide antibiotics showed the potential to cause dysbiosis in the gut	[58,59]
DNA replication inhibitors, or DNA-damaging antibiotics	Nitroimidazole; Quinolone; Nitrofuran	Treatment constantly changed microbial community compositions in the gut	[60,61,62]
Transcription and protein synthesis inhibitors	Aminoglycoside; Macrolides; Lincosamide; Tetracycline; Rifamycin;	Changes in the microbiome may result in further distress of the gut microbiome network; due to the non-antimicrobial effects of macrolides, changes include altered mucus secretion, ion transport, and inflammatory responses	[63,64,65]
Combination antibiotic treatment	Beta-lactam-Macrolide; Beta-lactam-Nitrofuran; Beta-lactam- Nitroimidazole; Beta-lactam-Macrolide Nitroimidazole; Glycopeptide- Nitroimidazole- Quinolone	Beta-lactam and glycopeptide antibiotics showed the potential to cause dysbiosis in the gut	[58,59]
Nitroimidazole	Metronidazole; Azithromycin	Decreased overall diversity and increased abundance of *Lactobacillus iners* in both the vaginal and urinary microbiomes	[66,67]

## Data Availability

Not applicable.
